# Role of CTX-M-15 gene in spread of extended-spectrum beta-lactamases among immunocompetent patients in Ghana

**DOI:** 10.4102/ajlm.v12i1.2135

**Published:** 2023-11-20

**Authors:** Noah Obeng-Nkrumah, Gloria D. Tawiah-Abrokwa, Enid Owusu, Francisca Duah, Daniel Oduro-Mensah, Paul Kwao, Bako Evariste, Appiah-Korang Labi

**Affiliations:** 1 Department of Medical Laboratory Science, School of Biomedical and Allied Health Sciences, University of Ghana, Accra, Ghana; 2 Department of Biochemistry, Cell and Molecular Biology, School of Biological Sciences, University of Ghana, Accra, Ghana; 3 Tenkodogo University Center, Thomas Sankara University, Ouagadougou, Burkina Faso; 4 Department of Medical Microbiology, University of Ghana Medical School, University of Ghana, Accra, Ghana

**Keywords:** extended-spectrum beta-lactamases, prevalence, inpatient, faecal carriage, Enterobacterales, Ghana

## Abstract

**Background:**

Patients with faecal carriage of extended-spectrum beta-lactamases (ESBL)-producing Enterobacterales serve as reservoirs and sources of dissemination and infection.

**Objective:**

This report examined immunocompetent patients for faecal carriage of ESBL-producing Enterobacterales in a district care hospital setting in Ghana.

**Methods:**

Between March 2019 and May 2020, cross-sectional sampling was performed to enrol patients and conduct questionnaire-structured interviews for factors that predispose patients to ESBL faecal carriage. Faecal samples from study patients were quantified for ESBL-producing Enterobacterales. The ESBL genes were characterised by polymerase chain reaction and sequencing.

**Results:**

The overall proportion of ESBL faecal carriage was 35.5% (*n* = 38/107). The *bla*_CTX-M_ gene, mostly CTX-M-15, was detected in 89.5% (*n* = 34/38) of the ESBL-producing isolates. The other ESBL types included *bla*_SHV_ (*n* = 3) and *bla*_OXA_ (*n* = 1). The CTX-M-15-positive isolates, when present in a faecal sample compared to the non-ESBL-CTX-M-15 isolates, constituted the predominant faecal Enterobacterales, with significantly higher colony counts than all other enterobacteria in that sample. In multivariate regression, independent risk factors for faecal carriage of ESBL-producing Enterobacterales were hospitalisation in the past year, infections since admission, use of antibiotics in the past 6 weeks, and admission from another hospital.

**Conclusion:**

The study found that CTX-M-15-producing isolates were the predominant faecal Enterobacterales, and that further investigations are needed to determine the reasons behind this dominance.

**What this study adds:**

The CTX-M-15-producing isolates dominance in this study shows the misuse and abuse of antibiotics in an African medical facility and indicates the potential role of immunity in controlling ESBL spread, which is to be investigated further.

## Introduction

The presence of extended-spectrum beta-lactamases (ESBL) in Enterobacterales remains the chief resistance mechanism against beta-lactam antibiotics. The ESBL-producing organisms may be widespread in Ghana. About 40% of the *Escherichia coli* and *Klebsiella pneumoniae* isolates causing infection in the nation’s largest tertiary care facility were found to be ESBL- producing.^1^ Similar prevalence rates have been reported elsewhere across the country. Infections with ESBL-producing Enterobacterales increase the risk of antibiotic treatment failure, morbidity and mortality, length of hospital stay, and cost of hospitalisation.^2,3^

Prior intestinal colonisation with ESBL-producing isolates has been identified as a significant risk factor for ESBL infections.^4,5^ These colonised patients remain reservoirs and serve as revolving doors for the spread of resistant pathogens between the community and hospital.^4,5^ This phenomenon increases the hospital’s potential as a repository of multidrug-resistant pathogens, with consequential increases in hospital-acquired infections and its associated economic burden to the patient, hospital and country.^2,3^ Despite this menace, few studies have reported on the intestinal carriage of ESBLs in Africa,^6,7,8,9,10,11,12,13,14^ particularly in Ghana.^15^ Many advances for reducing the ESBL menace are proposed in the literature. One such approach involves reducing the effect of patient characteristics that may predispose them to intestinal colonisation with ESBLs.^16,17^ An important factor that predisposes patients to ESBL intestinal colonisation is the misuse and abuse of antibiotics.^2,18^ This is more pronounced in immunosuppressed patients where antibiotics are systematically used as prophylaxis to prevent infections. However, there is a knowledge gap about correlations between immunosuppression, immunocompetence, and ESBL intestinal colonisation. Most studies on ESBL colonisation have looked at all patients together, without separating them into groups based on their immune status.^4,6,7,8,9,10,11,12,13,14,15^ This current study focuses specifically on the occurrence of intestinal colonisation with ESBL-producing Enterobacterales among immunocompetent patients, with particular emphasis on the quantification of ESBL-producing isolates and genotypes of ESBLs. Risk factors for intestinal colonisation with ESBLs were also analysed.

The overarching goal of the current study is to improve our understanding of ESBL faecal colonisation by analysing immunocompetent patients exclusively. The present study is part of a larger explorative study on immunocompetence and ESBL intestinal colonisation. By focusing on this specific group, we hope to provide valuable insights into the dynamics of ESBL colonisation in a subset of patients who have intact immunity. Such knowledge will assist in the development of targeted interventions and strategies to control the spread of ESBL-producing bacteria, improve patient outcomes, and optimise antibiotic usage within this specific patient population.

## Methods

### Ethical considerations

The study was approved by the Ethics and Protocol Review Committee of the School of Biomedical and Allied Health Sciences, University of Ghana (approval number: SBAHS-MD./10551508/AA/5A/2016-2017). Participation in the study was entirely voluntary, and patients were under no obligation to take part. Participating patients provided written informed consent before their enrolment in the study. Interviews were done at the respondent’s bedside to ensure privacy. Faecal specimens were assigned arbitrary numbers. Data were aggregated and randomised with unique identification numbers so that no individual could be linked to the study information.

### Study design and setting

This was a cross-sectional study to document the presence of intestinal carriage with ESBL-producing Enterobacterales. Faecal specimens were collected, and a questionnaire-based data collection tool (Online Supplementary File 1) was used to conduct interviews at the time of sampling to evaluate patient characteristics that predispose patients to intestinal colonisation with ESBL-producing isolates. The study was conducted from March 2019 through May 2020 at Achimota District Hospital, Accra, Ghana. The hospital is a 100-bed-capacity primary care government facility with no intensive care units.

### Study participants and sampling

Before the commencement of the study, appropriate permissions were sought from the hospital authorities. On day 1 of the survey, all patients hospitalised for ≥ 2 days at the Achimota District Hospital were considered potential study participants. An Android app called ‘Random Generator’ (Apps n Blue, Amman, Jordan) was used to randomly select hospitalised patients to be invited to participate in the study. We assigned each patient a number and entered them into the application. The application generated a random list of numbers, and we approached patients in the order listed. If a patient declined to participate, we moved on to the next patient on the list. On day 2, the patients previously contacted were requested to join the study. Those who agreed to participate were asked to provide informed consent. Participant’s medical records were examined with the help of an attending physician to identify patients who were immunocompetent, based on the definitions published by Public Health, England, on immunisation against infectious diseases.^19^ Patients were considered immunocompetent, in consultations with their attending physician, if they had no major organ or bone marrow transplant, no diagnosis of HIV, no severe combined immunodeficiency, no Wiskott-Aldrich syndrome, and no record of prescription of an immunosuppressive drug or steroid in the month before the sampling date.

### Data collection tool

For study participants, a structured data collection instrument (Online Supplementary File 1) was administered to collect demographic, clinical, lifestyle and hospitalisation history information. The tool was adapted from similar studies on ESBL faecal carriage risk factors reported in the literature.^2,16,18^ We collected data from multiple sources including medical records, direct interactions with the patients, and discussions with their attending doctor. The collected data included patients’ demographics (e.g., age, gender, employment status, educational level), which were primarily obtained from medical records. We determined the duration of hospital stays until the survey day through direct patient interactions. Information regarding the type of hospitalisation (hospitalised directly from home or transferred from another health facility) was gathered from discussions with the attending doctors. The number of patients in the participants’ ward at the time of recruitment was determined by investigators. Patient lifestyle characteristics, including the use of hand sanitiser at least once in the past three months, travel outside Ghana in the past year, travel outside the home in the past year, and access to pipe-borne water (treated municipal water source) in the household were collected through patient interviews. Information regarding toilet facilities in the household and animal contact in the past three months was also obtained through direct interactions with patients. Data on patient hospitalisation history, including hospitalisation in the past year, use of medication that affects intestinal flora, use of antibiotics in the past six months, and bacterial infections since admission, were extracted from the patients’ medical records, discussions with attending doctors, and, where applicable, interactions with patients.

### Culture and identification

Patients were given sterile stool containers and instructed to self-collect and submit at least 1 g of stool sample. Participant faecal samples were transported on ice at 0 °C to the microbiology laboratory. At the laboratory, 1 g of each faecal specimen was vortexed in 10 mL of 0.9% sterile saline. Ten-fold serial dilutions of each suspension were prepared at 10^−1^ to 10^−4^. The serial dilutions were cultured onto Statens Serum Institut enteric age (SSI Diagnostica, Hillerød, Denmark) by mixing 1 mL of each dilution with 24 mL of molten Statens Serum Institut agar (at 52 °C) and incubating aerobically at 35 °C – 37 °C for 18 h – 24 h. The Statens Serum Institut enteric medium combines selective properties with growth differentiation for direct isolation and rapid diagnosis of Enterobacterales. Quantification was performed by counting growing colonies per enterobacterium morphotype and estimating the number of colony-forming units per gram (CFU/g) of faecal sample. The number of Enterobacterales harboured by each faecal specimen in CFU/g was calculated as the number of colonies per morphotype × 10^1^ (for first faecal dilution) × 10^(dilution factor)^. Each Enterobacterales morphotype was subcultured onto agar for purity.

### Phenotypic determination of extended-spectrum beta-lactamases production

Each colony morphotype of Enterobacterales was examined for ESBL by the combined disk synergy assay. This test was performed using cefpodoxime (30 µg), ceftazidime (30 µg), cefotaxime (30 µg) and cefepime antibiotic discs (30 µg), with and without clavulanate (10 µg), on cation balanced Mueller-Hinton Agar. According to the Clinical and Laboratory Standards Institute interpretative guideline reference,^20^ the study isolates that demonstrated clavulanic acid effect defined by an increase in zone diameter greater than 5 mm for at least one test antibiotic were considered ESBL producers. *Klebsiella pneumoniae* ATCC 700603 (Becton Dickinson, Berkshire, United Kingdom) was used as a positive control for ESBL production. *Escherichia coli* ATCC 25922 (Becton Dickinson, Berkshire, United Kingdom) was used as a negative control. Only isolates with ESBL producer phenotypes were identified to the species level using the biochemical test kit MINIBACT-E diagnostic (SSI Diagnostica, Hillerød, Denmark), according to the manufacturer’s guidelines.

### Multiplex polymerase chain reaction for extended-spectrum beta-lactamases genes

Bacterial template DNA for polymerase chain reaction (PCR) was obtained by the boiling suspension method using distilled water as resuspension buffer.^21^ For the detection of ESBL genes, primers for the sulfhydryl variable active site enzyme (SHV), temoniera enzyme (TEM), oxacillinase 2 and 10 enzymes (OXA-2, OXA-10), and the cefotaxime-Munich (CTX-M) groups 1, 2, and 9 enzymes were used (Online Supplementary File 2). Each PCR mixture was a total of 24.5 µL, constituted as 2 µL template DNA, 12.5 µL of 2 × Multiplex Mastermix (Inqaba Biotec, Pretoria, South Africa), 2.5 µL of 10 × reverse or forward primer each, and 5 µL of DNAse/RNAse free water (Inqaba, Pretoria, South Africa). The PCR conditions^15,22^ were initial denaturation at 94 °C for 15 min followed by 30 cycles of amplification for TEM, SHV, OXA-1 and OXA-2 and 27 cycles of amplification for CTX-M group 1, 2, 9 consisting of 30 sec at 94 °C, 1 min at the appropriate annealing temperature for the specific primer (Online Supplementary File 1) and 1 min at 72 °C for primer extension, followed by 10 min at 72 °C for the final extension. The PCR products were analysed on 2% agarose gel (SeaKem^®^GTG^®^Agarose, Lonza, Basel, Switzerland) alongside a GeneRuler 100 base pairs DNA Ladder Plus marker (Fermentas, Leon-Rot, Germany).

### Nucleotide sequencing

All PCR products of TEM, SHV, CTX-M-1, CTX-M-2, CTX-M-8 and CTX-M-9 were sequenced to characterise specific genotypes of ESBL. The PCR products were first purified using NucleoFast 96 PCR plates (Macherey-Nagel GmbH & Co., Düren, Germany). The purified products, together with additional internal primers for CTX-M-1, CTX-M-9, SHV, and TEM (Online Supplementary File 2), were submitted on ice to Inqaba Biotec (Pretoria, South Africa) for nucleotide sequencing. The sequenced contigs were analysed using CodonCode Aligner version 10.0 (CodonCode Corporation, Centerville, Massachusetts, United States) and the aligned nucleotide sequences were compared by BLAST (https://blast.ncbi.nlm.nih.gov/Blast.cgi) with sequences in the The National Center for Biotechnology Information database (https://www.ncbi.nlm.nih.gov/). All TEM and SHV beta-lactamase sequences were compared to wild-type *E. coli* TEM-1 and SHV-1 sequences (Gen Bank accession number AF427133.1 and AF148850, respectively, at http://www.lahey.org/studies).

### Statistical analysis

Data were analysed using Statistical Package for Social Sciences version 16 (SPSS Inc., Chicago, Illinois, United States, 2007). Comparisons between categorical data were conducted with χ^2^ or Fisher’s exact tests. Continuous data were compared using a Student’s *t*-test. Point estimates of statistical significance were indicated with two-tailed *p* < 0.05. Univariate analyses were computed with an odds ratio (OR) with a 95% confidence interval (CI); variables with *p* < 0.05 were analysed in multivariate logistic regression models to determine independent associated predictor variables(s). The predictive accuracy of the models was evaluated by Hosmer and Lemeshow goodness-of-fit test (http://vassarstats.net/) with *p* < 0.05 suggesting that the model predicts accurately on average. The area under the Receiver Operating Characteristic Curve > 0.7 was used to analyse the discriminatory capability of ESBL faecal carriage versus their respective controls.

## Results

### Demographics of study patients

**FIGURE 1:**
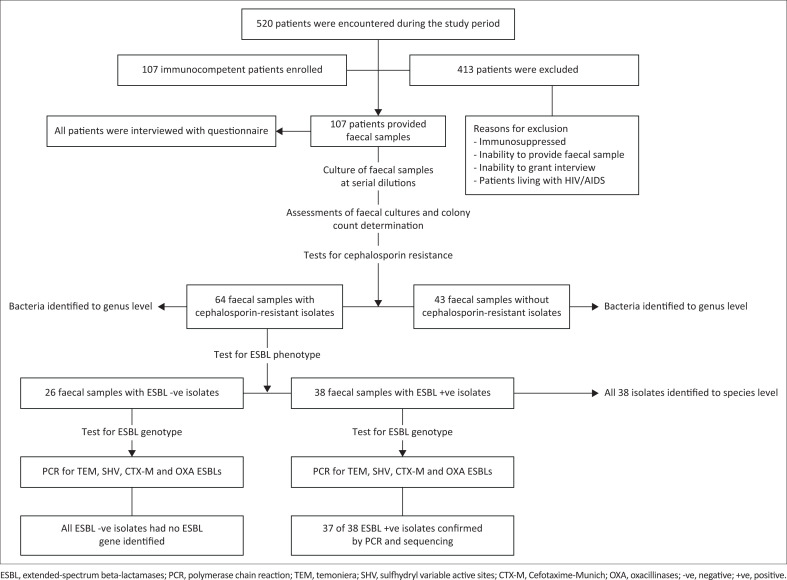
Design of study of faecal carriage of extended-spectrum beta-lactamases-producing Enterobacterales in Accra, Ghana, March 2019 to May 2020.

A total of 107 hospitalised patients with the age range of 13–38 years were enrolled (Figure 1). The mean age (with standard deviation) of the participants was 39.4 ± 13.8 (95% CI: 42–37). Based on medical records, 42 (39.2%) study patients were men and 65 (60.7%) were women. Of the 107 patients, 72 (67.3%) were admitted from home and 35 (32.7%) from another hospital (*n* = 35, 32.7%). Overall, 77 (72.0%) patients were employed, 6 (5.6%) had no formal education, and 64 (59.8%) had travelled outside Ghana in the past year. Of the 107 patients, 75 (70.1%) had had a previous hospitalisation in the past year, 61 (57.0%) had no pipe-borne water in their household, and 72 (43.0%) had no toilet facilities in their household. All 107 study participants submitted faecal specimens for laboratory investigations.

### Enterobacterales isolates cultured from faecal samples

A total of 676 Enterobacterales isolates were found in 107 faecal samples (Table 1). Out of these, 83 (77.5%) samples contained Enterobacterales that were resistant to either the third-generation cephalosporins (cefpodoxime, cefotaxime, ceftazidime) or the fourth-generation cephalosporin (cefepime), or both. These resistant isolates were referred to as third/fourth-generation cephalosporin-resistant isolates and included different species: *E. coli* (*n* = 37), *K. pneumoniae* (*n* = 12), other *Klebsiella* species (*n* = 10), *Citrobacter* species (*n* = 9), *Proteus* species (*n* = 4), *Providencia* species (*n* = 4), and *Morganella* species (*n* = 4). Among the third/fourth-generation cephalosporin-resistant isolates, 38 exhibited the ESBL producer phenotype, with *E. coli* (*n* = 31, 44.5%) and *K. pneumoniae* (*n* = 7, 8.4%) being the most common. Among these 38 isolates, 37 carried one or more ESBL genes. Overall, the prevalence of intestinal colonisation with ESBL-producing *E. coli* or *K. pneumoniae* was 35.5% (*n* = 38/107).

**TABLE 1:** Enterobacterales cultured from faecal samples in Accra, Ghana, March 2019 to May 2020.

Type and number of Enterobacterales	Number of faecal samples with					Number with confirmed ESBL gene	
	Growth	Cephalosporin-resistant isolates		ESBL phenotype			
		*n*	%	*n*	%	*n*	%
*Escherichia coli* (*n* = 161)	107	37	-	31	-	30	-
*Klebsiella pneumoniae* (*n* = 111)	71	12	-	7	-	7	-
Other *Klebsiella* species (*n* = 72)	21	10	-	0	-	0	-
*Citrobacter* species (*n* = 64)	63	9	-	0	-	0	-
*Enterobacter* species (*n* = 164)	32	4	-	0	-	0	-
*Providencia* species (*n* = 42)	32	4	-	0	-	0	-
*Morganella* species (*n* = 22)	11	4	-	0	-	0	-
*Serratia* species (*n* = 12)	9	4	-	0	-	0	-
*Proteus* species (*n* = 6)	6	1	-	0	-	0	-
Others (*n* = 22)	8	0	-	0	-	0	-
**Total faecal samples**	**107**	**83**	**77.5**	**38**	**35.5**	**37**	**34.6**

Note: Other faecal enterobacteria encountered in smaller numbers included *Edwardsiella* species (*n* = 6), *Erwinia* species (*n* = 5), *Hafnia* species (*n* = 4), *Shigella* species (*n* = 4), *Salmonella* species (*n* = 3).

ESBL, extended-spectrum beta-lactamases.

### Extended-spectrum beta-lactamases gene type

All isolates with ESBL phenotype harboured at least one corresponding ESBL gene type, except for one *E. coli* strain (which harboured a *bla*_TEM-13_ but no identifiable TEM, CTX-M, SHV or OXA ESBL gene). Nucleotide sequence analysis of CTX-M, SHV, TEM and OXA genes are shown in Table 2. Overall, 23 (54%) isolates harboured only one ESBL gene, 8 (25%) had two ESBL genes, while 6 (54%) combined an ESBL plus a broad-spectrum beta-lactamase gene. Eighteen different ESBL gene sequences (12 CTX-M types; 3 SHV types; 2 TEM types; and 1 OXA type) were identified either alone (*n* = 12) or in various combinations (*n* = 6). The most predominant ESBL gene type was *bla*_CTX-M-15_ (*n* = 12), followed by *bla*_CTX-M-2_ (*n* = 4) and *bla*_CTX-M-14_ (*n* = 4). Overall, 34 (91.8%) of the 37 ESBL sequenced genes were *bla*_CTX-M_ (21.6%, *n* = 8/37), which were found in various combinations with *bla*_SHV_ or *bla*_TEM_ or *bla*_OXA_ ESBLs. The SHV ESBLs were *bla*_SHV-40_, *bla*_SHV-86_, and *bla*_SHV-2A_; these were identified among only *K. pneumoniae* isolates. The TEM ESBLs were *bla*_TEM-3_ and *bla*_TEM-15_. The only OXA ESBL identified was *bla*_OXA-2_.

**TABLE 2:** Specific types of extended-spectrum beta-lactamases gene sequences in *Escherichia coli* and *Klebsiella pneumoniae* in Accra, Ghana, March 2019 to May 2020.

Type of cephalosporin resistance	ESBL gene†	*Escherichia coli*	*Klebsiella pneumoniae*	All
One ESBL type	SHV-40	0	1	1
	SHV-86	0	1	1
	TEM-3	1	0	1
	CTX-M-2	1	0	1
	CTX-M-3	1	-	1
	CTX-M-5	1	-	1
	CTX-M-14	4	-	4
	CTX-M-15	8	-	8
	CTX-M-20	1	-	1
	CTX-M-27	-	1	1
	CTX-M-31	1	1	2
	CTX-M-57	1	-	1
Total	-	19	4	23
Two ESBL types	CTX-M-1/SHV-40	-	1	1
	CTX-M-2/SHV-2A	-	1	1
	CTX-M-2/TEM-3	1	-	1
	CTX-M-3/TEM-15	1	-	1
	CTX-M-15/TEM-3	1	-	1
	CTX-M-15/TEM-15	1	-	1
	CTX-M-27/OXA-2	-	1	1
	CTX-M-57/TEM-3	1	-	1
Total	-	5	3	8
ESBL + broad beta-lactamases	CTX-M-2, TEM-1	1	-	1
	CTX-M-9, TEM-1	2	-	2
	CTX-M-12, TEM-1	1	-	1
	CTX-M-15, TEM-1	2	-	2
Total	-	6	0	6
Grand total	-	30	7	33
Only broad beta-lactamases	TEM-13	1	-	1

ESBL, extended-spectrum beta-lactamases.

† , TEM-1 and TEM-13 are not ESBL gene sequence types.

### Faecal load of extended-spectrum beta-lactamases producers

The faecal concentration of ESBL-positive *E. coli* and *K. pneumoniae* varied considerably by the type of ESBL gene present (Table 3). For example, among patients (*n* = 11) colonised by a CTX-M-15-positive *E. coli* and/or *K. pneumoniae*, the ESBL producers comprised > 80.0% of the total Enterobacterales counts per gram of the faecal sample (mean ± standard deviation [s.d.]: 190 × 10^4^ ± 27 × 10^4^ CFU/g; interquartile range [IQR]: 175 × 10^4^ – 205 × 10^4^ CFU/g). In contrast, the faecal concentration of ESBL-negative Enterobacterales was ≤ 50 × 10^4^ CFU/g per patient (for all 11 patients: mean ± s.d.: 38 × 10^4^ ± 6 × 10^4^ CFU/g; IQR: 34 × 10^4^ – 43 × 10^4^ CFU/g). We then compared the faecal load of Enterobacterales among the 27 patients with non-CTX-M-15 ESBL colonisation. Within this cohort, the ESBL-negative Enterobacterales constituted the predominant (> 80%) faecal isolates (for all 27 patients: mean ± s.d.: 134 × 10^4^ CFU/g; IQR: 46 × 10^4^ – 200 × 10^4^ CFU/g). The mean faecal concentration of non-CTX-M-15 ESBL-positive *E. coli* and/or *K. pneumoniae* in all 27 patients was 22 × 10^4^ ± 21 × 10^4^ CFU/g with an IQR of 10 × 10^4^ – 24 × 10^4^ CFU/g. The total faecal concentration of the non-CTX-M-15 *E. coli* and *K. pneumoniae* comprised 16.4% of the overall faecal concentration of the Enterobacterales among the patients with non-CTX-M-15 ESBL colonisation.

**TABLE 3:** Comparison of the faecal concentration (colony-forming units/g) of extended-spectrum beta-lactamase-positive and all extended-spectrum beta-lactamase-negative Enterobacterales bacteria in faecal samples of 38 extended-spectrum beta-lactamase faecal carriers in Accra, Ghana, March 2019 to May 2020.

Patient identity		Type of ESBL faecal carriage	Bacteria load (concentration × 10^4^ CFU/g [%])						
			Total	Isolates with CTX-M-15 ESBLgene†		Isolates with other ESBL gene but no CTX-M-15		ESBL-negative isolates	
				*n*	%	*n*	%	*n*	%
1	Patient 2	*E. coli* CTX-15/TEM-1	183	150	81.9	-	-	33	18.0
2	Patient 4	*E. coli* CTX-15	197	162	82.2	-	-	35	17.7
3	Patient 8	*E. coli* CTX-15	212	171	80.6	-	-	41	19.3
4	Patient 12	*E. coli* CTX-15/TEM-1	222	178	80.2	-	-	44	19.8
5	Patient 14	*E. coli* CTX-15	216	181	83.7	-	-	35	16.2
6	Patient 16	*E. coli* CTX-15	229	189	82.5	-	-	40	17.4
7	Patient 19	*E. coli* CTX-15	224	190	84.8	-	-	34	15.2
8	Patient 20	*E. coli* CTX-15	236	191	80.9	-	-	45	19.1
9	Patient 24	*E. coli* CTX-15	270	220	81.5	-	-	50	18.5
10	Patient 26	CTX-M-15/TEM-3	262	230	87.7	-	-	32	12.2
11	Patient 29	*E. coli* CTX-M-15/TEM-15	266	236	88.7	-	-	30	11.3
12	Patient 33	*E. coli* CTX-M-14	290	-	-	12	4.1	278	95.9
13	Patient 37	*K. pneumoniae* SHV-86	280	-	-	10	3.5	270	96.4
14	Patient 39	*E. coli* TEM-3	159	-	-	7	4.4	152	95.6
15	Patient 41	*E. coli* CTX-M-2	283	-	-	12	4.2	271	95.7
16	Patient 42	*E. coli* CTX-M-3	183	-	-	21	11.5	162	88.5
17	Patient 43	*E. coli* CTX-M-5	256	-	-	56	21.9	200	78.1
18	Patient 47	*E. coli* CTX-M-14	223	-	-	12	5.3	211	94.6
19	Patient 50	*E. coli* CTX-M-15/TEM-3	181	-	-	10	5.5	171	94.7
20	Patient 52	*K. pneumoniae* CTX-M-27/OXA-2	109	-	-	9	8.3	100	91.7
21	Patient 54	*E. coli* CTX-M-15/TEM-15	207	-	-	7	3.3	200	96.6
22	Patient 58	*K. pneumoniae* SHV-40	88	-	-	10	11.3	78	88.6
23	Patient 65	*E. coli* CTX-M-12/TEM-1	103	-	-	11	10.6	92	89.3
24	Patient 73	*E. coli* CTX-M-9/TEM-1	230	-	-	21	9.1	209	90.8
25	Patient 77	*E. coli* CTX-M-14	79	-	-	23	29.1	56	70.8
26	Patient 81	*E. coli* CTX-M-14	122	-	-	45	36.9	77	63.1
27	Patient 85	*E. coli* CTX-M-20	147	-	-	67	45.6	80	54.4
28	Patient 91	*K. pneumoniae* CTX-M-27	301	-	-	100	33.2	201	66.7
29	Patient 93	*K. pneumoniae* CTX-M-31	294	-	-	7	2.3	287	97.6
30	Patient 97	*E. coli* CTX-M-57	170	-	-	10	5.8	160	94.1
31	Patient 98	*E. coli* CTX-M-20	145	-	-	24	16.5	121	83.4
32	Patient 100	*E. coli* CTX-M-57/TEM-3	189	-	-	33	17.4	156	82.5
33	Patient 101	*K. pneumoniae* CTX-M-1/SHV-40	123	-	-	20	16.3	103	83.7
34	Patient 103	*E. coli* CTX-M-3/TEM-15	140	-	-	34	24.3	106	75.7
35	Patient 104	*E.coli* CTX-M-9/TEM-1	297	-	-	10	3.4	287	96.6
36	Patient 105	*E. coli* CTX-M-2/TEM-3	261	-	-	11	4.2	250	95.7
37	Patient 106	*E. coli* CTX-M-2/TEM-1	86	-	-	9	10.5	77	89.5
38	Patient 107	*K. pneumoniae* CTX-M2/SHV-2A	309	-	-	9	2.9	300	97.1

Note: Isolates with CTX-M-15 ESBL gene (1–11) – Mean ± s.d.: 190 ± 27, IQR: 175–205; ESBL-negative isolates (1–11) – Mean ± s.d.: 38 ± 6, IQR: 34–43. Isolates with other ESBL gene but no CTX-M-15 (12–38) – Mean ± s.d.: 22 ± 21, IQR: 10–24; ESBL-negative isolates (12–38) – Mean ± s.d.: 134 ± 89, IQR: 46–200.

s.d., standard deviation; IQR, interquartile range; ESBL, extended-spectrum beta-lactamases; %, percent of the total faecal enterobacteria colony counts; SHV, sulfhydryl variable active sites; TEM, temoniera; OXA, oxacillinases; CTX-M, Cefotaxime-Munich; s.d., standard deviation; IQR, interquartile range; CFU, colony-forming units.

† , For only *E. coli* and *K. pneumonia*.

### Univariate comparison of patients’ characteristics

Compared to those admitted from home, patients who were admitted from another hospital had a statistically significant higher likelihood to have faecal carriage with ESBL-producing *E. coli* or *Klebsiella pneumoniae* (OR: 3.5; 95% CI: 1.5–8.4; *p* = 0.003) (Table 4). Patients with a history of hospitalisation in the preceding year were frequently faecal carriers of ESBL producers (OR: 3.6; 95% CI: 1.5–8.6; *p* = 0.003). Faecal carriage of ESBL producers had a statistically significant association with patients who had acquired an infection since admission (OR: 5.2; 95% CI: 2.2–12.3; *p* < 0.001). Lifestyle patterns that had a statistically significant association with faecal carriage of ESBL producers included chronic alcohol use (OR: 12.7; 95% CI: 4.9–32.8; *p* < 0.001) and animal contact in the preceding 3 months (OR: 3.7; 95% CI: 1.5–8.5; *p* < 0.002). Diarrhoea (OR: 0.1; 95% CI: 0.02–0.16; *p* < 0.001) or use of hand sanitiser in preceding three months (OR: 0.4; 95% CI: 0.1–2.0; *p* < 0.04) indicated a protective effect.

**TABLE 4:** Univariate comparison of the risk factors among patients with and without ESBL-positive faecal carriage in Accra, Ghana, March 2019 to May 2020.

Patient demographics, lifestyle, hospitalisation and clinical characteristics	Patients with ESBL faecal carriage (*n* = 38)	Patients without ESBL faecal carriage (*n* = 69)	Crude odds ratio	95% CI	*p*
**Patient demographics**					
Age (Mean ± s.d.)	39.6 ± 13.8	39.5 ± 13.8	-	-	0.13
**Age group**					
Neonates (< 28 days)	0	0	0	-	-
Infants (28 days – 5 years)	0	0	0	-	-
Paediatric (> 5 years – 18 years)	1	1	1.8	0.1–30.2	1.00
Adults (> 18 years – 65 years)	36	62	1.9	0.3–10.0	0.54
Elderly (> 65 years)	1	6	0.3	0.3–2.5	0.42
**Gender**					
Male	15	27	1.0	0.4–2.3	1.00
Female	23	42	2.0	0.4–2.2	1.00
**Employed**	30	47	1.7	0.2–1.4	0.20
**Formal education**	36	65	0.4	0.3–0.5	0.13
**Type of formal education**					
Primary	10	30	0.4	0.1–1.1	0.08
Secondary	14	23	1.2	0.5–2.7	0.71
Tertiary	12	12	2.1	0.8–5.5	0.09
**Lifestyle factors**					
Used hand sanitiser in past 3 months	6	24	0.4	0.1–2.0	0.04
**Frequency of hand sanitiser use per day**					
1	0	11	-	-	0.06
2	2	4	1.6	0.2–11.4	0.51
3	1	5	0.8	0.1–8.1	1.00
4	2	3	3.5	0.4–28.1	0.50
> 4	1	1	4.6	0.2–86.6	0.33
**Daily handwashing in past month**	38	43	-	-	-
**Frequency of hand washing per day**					
1	6	13	0.8	0.3–2.3	0.73
2	9	5	4.0	1.2–13.0	0.02
3	8	11	1.4	0.5–4.0	0.51
4	11	6	0.7	0.3–1.6	0.42
> 4	4	14	0.5	0.1–1.5	0.24
**Chronic smoking**	2	4	0.9	0.2–5.2	1.00
**Chronic alcohol use**	29	14	12.7	4.9–32.8	< 0.001
**Travelled overnight outside home in past year**	26	43	1.3	0.5–3.0	0.52
**Travelled outside Ghana in past year**	10	12	1.6	0.6–4.4	0.34
**Animal contact in past 3 months**	20	16	3.7	1.5–8.5	0.002
**Pipe-borne water in household**	19	30	1.3	0.5–2.8	0.52
**Toilet in household**	30	54	1.0	0.3–2.7	0.94
**Hospitalisation history and clinical characteristics**					
Number of patients in hospital ward	8.3 ± 2.8	8.5 ± 2.5	0.7	-	-
Total duration of hospital stays	2.9 ± 1.4	3.7 ± 8.6	-	-	0.63
**Admitted from**	-	-	-	-	-
Hospital	18	14	3.5	1.5–8.4	0.003
Home	20	55	-	-	-
**Infection since admission**	24	17	5.2	2.2–12.3	< 0.001
**Surgery since admission**	11	14	1.6	0.6–4.0	0.31
**Functional status (need help of any sort)**	9	9	2.1	0.7–5.7	0.20
**Co-morbidities**					
Respiratory infections	5	3	3.3	0.7–14.8	0.14
Diarrhoea	8	56	0.1	0.02–0.16	< 0.001
Diabetes	7	12	1.1	0.38–3.0	0.88
**Hospitalised in past year**	28	30	3.6	1.5–8.6	0.003
**Invasive procedure of any type in past year**	4	3	2.6	0.5–12.2	0.24
**Presence of indwelling catheter**	24	26	2.8	1.2–6.5	0.01
**Use of medications that affect intestinal flora†**	18	22	1.9	0.8–4.3	0.12
**Used antibiotic in last 6 months**	24	23	3.4	1.5–7.8	0.002
**Used antibiotic without prescription**	17	19	2.1	0.9–4.9	0.07
**Current antibiotic use**	27	47	1.1	0.4–2.7	0.75
**Specified antibiotics used**					
Aminoglycosides	4	10	0.7	0.2–2.4	0.76
Beta-lactam	14	27	0.9	0.4–2.1	0.82
Beta-lactam/aminoglycosides	2	4	0.9	0.2–5.2	1.00
Beta-lactam/fluoroquinolones	6	5	2.4	0.7–8.5	0.19
Beta-lactam /macrolides	2	2	1.9	0.3–13.8	0.61
Fluoroquinolones	2	8	0.4	0.08–2.1	0.32
Lincosamides	3	3	1.8	0.4–9.6	0.67
Macrolides	4	4	1.8	0.4–8.0	0.45
Metronidazole	2	2	1.8	0.3–13.8	0.61
Phenols	0	1	-	-	-
None	1	3	0.6	0.06–5.9	1.00

Note: Risk ratios and Fisher’s exact probability tests *p*-values were calculated for variables with cell counts < 5.

*N*, number; CI, confidence interval; ESBL, extended-spectrum beta-lactamases; s.d., standard deviation.

† , Use of stomach acids neutraliser, proton pump inhibitor, H2 blockers

### Multivariable logistic regression for independent risk factors

Use of antibiotics in the previous 6 months was the strongest predictor for faecal carriage of ESBL producers (adjusted OR: 3.4; 95% CI: 1.5–10.5; *p* = 0.0001) (Table 5). Patients with faecal carriage of ESBL producers had used antibiotics 3.4 times more during the preceding six months than patients without faecal carriage of ESBL producers (Table 5). Other independent risk factors were the acquisition of infection(s) since admission (adjusted OR: 3.2; 95% CI: 1.9–7.8; *p* = 0.002), hospitalisation in the preceding year (adjusted OR: 1.5; 95% CI: 1.2–3.6; *p* = 0.04), and admission from another hospital (adjusted OR: 1.6; 95% CI: 1.1–4.3; *p* = 0.003).

**TABLE 5:** Independent risk factors for faecal carriage with extended-spectrum beta-lactamase-producing Enterobacterales among study participants using multivariate logistic regression analysis in Accra, Ghana, March 2019 to May 2020.

Variable	Level	Adjusted OR	95% CI	*p*-value
Admitted from a hospital	Yes/No	1.6	1.1–4.3	0.003
Chronic alcohol use	Yes/No	2.4	0.9–5.3	0.08
Used hand sanitiser in past 3 months	Yes/No	0.7	0.1–2.0	0.07
Diarrhoea	Yes/No	0.9	0.3–1.6	0.11
Animal contact in past 3 months	Yes/No	1.7	0.8–3.5	0.09
Hospitalised in past year	Yes/No	1.5	1.2–3.6	0.04
Presence of indwelling catheter	Yes/No	1.5	0.7–5.5	0.24
Used antibiotic in last 6 months	Yes/No	3.4	1.5–10.5	< 0.001
Infection since admission	Yes/No	3.2	1.9–7.8	0.002

OR, odds ratio; CI, confidence interval.

## Discussion

In this present study, we examined the occurrence of faecal carriage of ESBL-producing Enterobacterales recovered from immunocompetent patients in a district hospital setting in Ghana. The overall prevalence of faecal carriage by ESBL-producing isolates was 35.5%. The CTX-M genes, mostly *bla*_CTX-M-15_, were the predominant ESBL genotype. When CTX-M-15-producing isolates occurred in a faecal specimen, these isolates were the predominant faecal Enterobacterales. In this study, independent risk factors for ESBL faecal carriage included previous hospitalisation for the past year, admission from another hospital, infections since admission, and use of antibiotics during the past six weeks.

Studies on ESBL faecal carriage among hospitalised patients is globally prevalent but there is little data on the role of immunity in this subject matter. Available studies on ESBL faecal colonisation have not clearly distinguished between immunocompetent and immunosuppressed patients. What is not clear in the literature is whether ESBL intestinal colonisation patterns differ in patients with varying immune statuses. Although answers to this conundrum are beyond the purview of this study, the findings of this work compared to other studies may offer some preliminary insights. According to a meta-analysis review conducted by Bezabih in 2021,^23^ the combined prevalence of ESBL-producing *E. coli* intestinal carriage in apparently healthy individuals across African communities was estimated to be 21.4% (95% CI: 12.7% – 30.1%). In contrast, our current study found a higher prevalence of 35.5% for all Enterobacterales among hospitalised patients. This suggests that the risk of ESBL carriage may be greater among hospitalised individuals compared to the general population in community settings. However, while the pooled prevalence provides a useful estimate of ESBL carriage across Africa, individual studies may reveal variations and provide more localised data because the prevalence of ESBL carriage varies widely between different countries, regions, and populations. For example, the ESBL faecal carriage prevalence in our current study is significantly lower than the ESBL levels reported for other patient hosts in Ghana by Obeng-Nkrumah et al. in 2013 (49.3%; *n* = 148/300),^1^ Feglo et al. in 2013 (48.4%, *n* = 77/159),^24^ Feglo and Adu-Sarkodie in 2016 (57.8%, *n* = 234/405),^25^ Falgenhauer et al. in 2019 (61%, *n* = 33/54),^26^ Donkor et al. in 2022 (44.6%, *n* = 145/334),^27^ and Mahazu et al. in 2022 (62.2%, *n* = 102/164).^28^ It is worth noting that variations in study populations can also influence ESBL carriage rates. For example, the proportion of ESBL faecal carriage reported in our work is lower than that cited for similar populations in comparative reviews^6,17,18^ spanning several regions across Africa, including Uganda in 2023 (61%),^29^ Ghana in 2022 (53.1%),^30^ Kenya in 2022 (44%),^31^ Niger in 2022 (92.4%),^32^ Malawi in 2021 (47.2%),^33^ Central African Republic in 2016 (59%),^10^ Morocco in 2014 (42.9%),^14^ and Cameroon in 2013 (55.3%).^11^ In all these studies, the reports were silent on whether the study patients were immunocompetent or immunosuppressed. Indeed, there are limited data on ESBL faecal carriage versus immunity and the paucity of data hinders the ability to compare our findings. Nonetheless, our report provides much-needed data on ESBL carriage rates in immunocompetent patients and serves as a valuable foundation for future studies aiming to compare data and explore the impact of immunity on ESBL carriage rates. This will help us to better understand the factors that contribute to ESBL carriage and to develop strategies for preventing and treating ESBL infections.

A noteworthy finding in this current study was the fact that whenever CTX-M-15 ESBL-positive isolates occurred in a faecal sample, these isolates constituted the predominant faecal bacteria compared to all other enterobacteria. In such instances, the CTX-M-15-producing isolates constituted over 80% of the total faecal enterobacteria. These patients with CTX-M-15 faecal carriage comprise high-density ESBL shedders and may be of public health significance in the dissemination of multidrug-resistant bacteria. Reports on CTX-M ESBLs, predominantly CTX-M-15, are increasingly becoming common worldwide. In the past decade, various studies conducted across Africa^7,17,18^ and various global settings^34,35^ consistently indicate that the CTX-M gene is the most prevalent among ESBL genes. Overall, CTX-M enzymes represented 91.8% of the ESBLs in our current study. This observation mirrors the current trend observed across Africa. In Ghana, a recent study was conducted across different communities to examine faecal samples from 736 healthy community residents in order to assess the occurrence of ESBL-producing Enterobacterales. The results showed that over 47% of the participants carried ESBL producers in their faecal specimens. Furthermore, 99.4% (*n* = 354/356) of the isolates with ESBL phenotype carried the *bla*_CTX-M_ gene, predominantly CTX-M-15. A possible explanation for the dominance of CTX-M genes could be its ability to localise on large plasmids and co-harbour other resistant genes such as *bla*_AmpC_, quinolone-resistance genes or methylase-affecting aminoglycosides.^23^ The CTX-M-15-borne plasmids in particular have been reported to have a high conjugation frequency and are thus more frequently disseminated to other Enterobacterales species.^24,25^ The CTX-M genes are transmissible by conjugation with high transfer frequencies of 10^−7^ to 10^−2^ per donor cell. Perhaps the few TEM, SHV and OXA ESBL types identified in this study point to the fact that CTX-M genes are fast replacing other ESBL types.^26^ Overall, our report broaches relevant conversations on the possible role of immunity to limiting the spread of ESBL. Will a dominant population of colonising non-ESBL Enterobacterales limit the intestinal occurrence and significance of CTX-M-15? Does the immune status of patients play a role in intestinal colonisation by CTX-M-15 and other ESBL types? Further investigations to ascertain the reasons behind such dominance is warranted. This report is the first of our series to compare ESBL carriage between immunocompetent and immunosuppressed patients.

In multivariable logistics analysis, this study identified antibiotic use in the past 6 months as the highest predictor of ESBL faecal carriage. Several other studies have linked prior antibiotic use within the past 4 or 12 months to an increased possibility of faecal ESBL colonisation.^2,5,15,16,18^ In our univariate comparisons, patients with ESBL faecal carriage were significantly associated with an acquired infection in the preceding 3 months. The analysis also showed that antibiotic use in the past 3 weeks among patients with ESBL faecal carriage was about four-fold higher compared to that of patients without ESBL faecal carriage. Some studies have even suggested that the more current the antibiotic use, the greater the risk of faecal carriage with an ESBL-positive enterobacteria.^5,17,18^ These observations are not surprising given that ESBLs and many other antibiotic-resistant mechanisms evolved as a consequence of the misuse and abuse of antibiotics.

### Limitations

There are some potential limitations of this study that should be discussed briefly. Bacterial clonal relatedness was not investigated in this work. Such investigations would have enabled the study to compare various ESBL faecal enterobacteria to determine the extent to which these isolates are being disseminated. Another noteworthy limitation is the rather few study patients. A more large-scale survey is much more likely to be with little bias for high-at-risk ESBL faecal carriers.

### Conclusion

The findings of this study suggest that faecal carriage of ESBL-producing Enterobacterales is a significant public health concern in Ghana. The high prevalence of ESBL-producing bacteria, the association with antibiotic use, and the identification of CTX-M-15 genes as a potential driver of multidrug-resistant bacteria dissemination in immunocompetent patients are all concerning trends. These findings highlight the need for increased surveillance of ESBL-producing bacteria in Ghana and other parts of Africa. Further research is needed to explore the relationship between ESBL faecal carriage and immunity, understand the dominance of CTX-M genes, and address the consequences of antibiotic misuse. More research is also needed to better understand the factors that contribute to ESBL faecal carriage and to develop effective interventions to prevent the spread of these bacteria.

## Supplementary Material


