# Supplementary material for: Role of CTX-M-15 gene in spread of extended-spectrum beta-lactamases among immunocompetent patients in Ghana
Source: Afr J Lab Med. 2023 Nov 20;12(1):2135. doi: 10.4102/ajlm.v12i1.2135 (PMC10696557; doi:10.4102/ajlm.v12i1.2135)
Supplement: Supplementary file 1 [file AJLM-12-2135-s001.pdf]

Note: This is Online Supplementary Document 1 of Obeng-Nkrumah N, Tawiah-Abrokwa GD, Owusu E, et al. Role of CTX-M-15 gene in spread of extended-spectrum beta-lactamases among immunocompetent patients in Ghana. Afr J Lab Med. 2023;12(1), a2135. https://doi.org/10.4102/ajlm.v12i1.2135

Supplementary data 1: Data collection tool (Accra, Ghana, 2023)

==

Data Collection Tool

Towards the determination of risk factors for ESBL faecal carriage

INTESTINAL CARRIAGE WITH EXTENDED-SPECTRUM BETA-LACTAMASE PRODUCING ENTEROBACTERIA INIMMUNOCOMPETENT PATIENTS

Participant Data Collection Instrument

Date:……..../……..../………… Site ……………………………

We want to ask you a few questions, your participation is voluntary and you may stop at any time while answering the questions. Many questions will assess your knowledge, behaviors, and the environment you live.

Patient code:

Q 1. Personal information:

**Q1.1**	Name:	
**Q1.2**	Date of birth:	Date of admission:
**Q1.3**	Gender:	Female:___ Male:____
**Q1.4**	Ward name:<br>Number of beds:<br>Number of patients in ward:<br>Number of healthcare staff:<br>Flush toilet in ward:<br><br>Indicate number	 ……………………………<br>……………………………<br>……………………………<br>……………………………<br>Yes ☐ No ☐: if 'No' indicate type…………………..<br>………………

	Source of water:	Running tap ☐ ; Veronica buckets ☐ ; Others (specify)………….
**Q1.5**	Admitted from home ☐ current hospital ☐ another healthcare facility ☐	
**Q1.6**	Where do you live currently? ______________________________<br>GPS Address ___________________<br>How long? _______________________	

Q2. Questions of underlying diseases:

Do you suffer from any of the following diseases?

Admitting diagnosis:		Yes:	No:	I do not know:
**Q2.1**	Haematological disorders:			
**Q2.2**	Respiratory infections:			
**Q2.3**	Diarrhoea:			
**Q2.4**	Diabetes:			
**Q2.5**	Cancer:			
**Q2.6**	HIV/AIDS:			
**Q2.7**	Liver cirrhosis:			
**Q2.8**	Alcoholism:			
**Q2.9**	Infections since admission			
**Q2.10**	Surgery since admission			
**Q2.11**	Functional status: Need help of any sort			
**Q2.12**<br><br><br><br><br><br>**Q2.13**	Immunosuppression (of any type)<br>Steroids<br>Chemotherapy in past 3 months<br>Radiation in past 3 months<br>Others (specify):……………………………………..<br><br>Gastro-oesophageal disease…………………………….			

Q3. Questions of underlying patient's lifestyle:

**Q3.1**	Alcohol hand rubs/sanitizer	Yes	No

	If yes, indicate how often you use this in a day:<br><br>Once☐ ; Twice ☐; Thrice ☐; > Thrice ☐ ; as often as possible ☐		
**Q3.2**	Do you wash your hands?<br><br>How often do you wash your hands.<br><br>Once ☐; Twice ☐ ; Thrice☐ ; > Thrice☐; as often as possible ☐	Yes	No
**Q3.3**	Employment	Yes	No
**Q3.4**	Current smoking	Yes	No
**Q3.5**	Alcoholism	Yes	No
**Q3.6**	Education<br><br>Indicate status: Primary☐; Secondary☐; Tertiary ☐ None ☐	Yes	No
**Q3.6**	Profession/Occupation:		
**Q3.6**<br><br>**Q3.7**<br><br>**Q3.8**<br><br>**Q3.9**<br><br>**3.10**	Have travel overnight outside your home in past one year................................<br><br>Have travel outside Ghana in the past year..<br><br>Number of person in the household...<br><br>Toilet facility in household..<br><br>Pipe borne water in household..		

Q3.11 Do you have pets? ☐ Yes ☐ No

Q3.12 If yes, which pets? ☐ Cat ☐ Dog ☐ Birds ☐Fish ☐ Other ___________________

Q3.13 How many? ___________________________

Q3.14 Do you have any other animals' aside pets? ☐ Yes ☐ No

Q3.15 If yes, which animals? ☐ Goat ☐ Sheep ☐ Cow ☐ Chicken ☐ Other ______

Q3.16 How many? ___________________________

**Q3.17** Do you swim? ☐ Yes ☐ No<br>**Q3.18** If yes, which waterbodies do you swim in? ___________________________
**Q3.19** How often do you come into contact with that waterbody in a week?<br>a. Once b. Twice c. Thrice d. More than three times
**Q3.20**. Do you handle raw meat? ☐Yes ☐No<br>**Q3.21** If yes, how often do you handle meat weekly?<br>a. Once b. Twice c. Thrice d. More than three times
**Q3.22** Do you handle raw fish? ☐Yes ☐No<br>**Q3.23** If yes, how often do you handle fish weekly?<br>a. Once b. Twice c. Thrice d. More than three times
**Q3.24** What are your sources of drinking water? ☐Tap water ☐Sachet water ☐ Bottled water ☐ Bore hole ☐ Other _____________________
**Q3.25** How do you store your drinking water? ☐ Freezing ☐ Barrel ☐ Bucket ☐ Poly tank ☐ Other________________________
**Q3.26** How do you store your bathing water? ☐ Barrel ☐ Bucket ☐ Poly tank ☐ Other________________________
**Q3.27**. Do you have any relative or close relation working in the health sector? ☐ Yes ☐ No

Q4.History of hospitalization

**Q.4.1**	Hospitalization in the past 1 year<br>Number of hospitalization………………………………………………<br>Total duration hospital stay ……………………………………………	Yes:	No:
**Q.4.2**	Invasive procedure of any type in past 1 year (endoscopy, gastroscopy, sigmoidoscopy, colonoscopy, etc)	Yes:	No:

**Q.4.3**	Presence of central vascular catheter	Yes	No
**Q.4.4**	Presence of peripheral vascular catheter<br>Presence of urinary catheter<br>Presence of intubation	Yes	No
**Q.4.5**	Use of medications that affect intestinal flora<br>Drugs that neutralize stomach acids<br>Proton pump inhibitors<br>H2 blockers	Yes	No
**Q.4.6**<br>**Q.4.7**<br>**Q4.8**	Used antibiotics in last 3 months<br>Used antibiotics without prescription<br>Indicate type of antibiotics used in last 3 months……………………..<br>Current antibiotics used…………………………………………………………<br>Specify antibiotics…………………………………………………………………..<br>Animal contact in the past 6 months………………………………………..	Yes	No

Comments:
……………………………………………………………………………………………
……………………………………………………………………………………………
……………………………………………………………………………………………
……………………………………………………………………………………………
……………………………………………………………………………………………
…………………………

Supplementary data 2. PCR primers and thermocycling conditions (Accra, Ghana, 2023)

Primer (target)	Primer sequences (5'-3')	T °C [a]	PCR cycles
TEM (918 bp)	FP: GTATCCGCTCATGAGACAATAACCCTG<br>RP: CCAATGCTTAATCAGTGAGGCACC<br>Internal FP CCGGAGCTGAATGAAGCCAT<br>Internal RPCGTTGTTGCCATTGCTGCAG	63°C at 90 seconds	30
SHV (842 bp)	FP: CGC CTG TGT ATT ATC TCC CTG TTAGCC<br>RP: TTG CCA GTG CTC GAT CAG CG<br>Internal FP : ACCATGAGCGATAACAGCGC<br>Internal RP: AAGCGCCTCATTCAGTTCCG	63°C at 90 seconds	30
OXA-2 (330 bp)	FP: GTTAACAGGGGCTTTGCAGG<br>RP: TGCACGCAGTATCCAGTTGC	63°C at 90 seconds	30
OXA-10 (655 bp)	FP: ATGAAAACATTTGCCGCATATGTA<br>RP: ACACCAGGATTTGACTCAGTTCC	63°C at 90 seconds	30
CTX-M-1(940 bp)	FP: GACAGACTATTCATGTTGTTGTTAWTTCG<br>RP: CCGTTTCCSCTATTACAAA<br>Internal FP: GGACGATGTCACTGGCTGAG<br>Internal RP: TTTCGTCTCCCAGCTGTCGGG	50 °C at 90 seconds	30
CTX-M-2 (253 bp)	FP: ACAGTTGGTGACGTGGCTTAAGG<br>RP: TCAGAAACCGTGGGTTACGA	50 °C for 90 seconds	30
CTX-M-8/25/26 (690/346bp)	FP1: ACATCGCGTTAAGCGGAT<br>FP2: GCACGATGACATTCGGG<br>RP: AACCCACGATGTGGGTAGC	50 °C for 90 seconds	30
CTX-M-9 (860bp)	FP: ATGGTGACAAAGAGAGTGCAACG<br>RP: ATGATTCTCGCCGCTGAAGC<br>Internal FP: CAAATTGATTGCCCAGCTCG<br>Internal RP: AAACGTCTCATCGCCGATCG	50 °C for 90 seconds	30

[a] T, Annealing temperature; °C, degree celsius.
